# Supplementary material for: Modeling epithelial deformation and cell rearrangement in response to external forces during Zebrafish epiboly
Source: NPJ Syst Biol Appl. 2026 Apr 21;12:63. doi: 10.1038/s41540-026-00708-0 (PMC13139408; doi:10.1038/s41540-026-00708-0)
Supplement: Supplementary file 1 — Supplementary Information [file 41540_2026_708_MOESM1_ESM.pdf]

Supplementary Note. Epibolic deformation of the zebrafish EVL is Convergent Extension

The term "convergent extension" has been used inconsistently in the literature; for the purposes of this study we adopt historical precedent[1–3] and define it as a tissue-level rather than a cell-level phenomenon; and as a tissue shape change rather than the cellular mechanisms underlying it; and therefore, not as a cause of morphogenesis, but as a macroscopic description of it. The two components of CE – convergence, and extension – can also be combined with deformation in the third dimension: either thinning or thickening (e.g. CE of the neurectoderm during gastrulation and neurulation in the frog *Xenopus laevis*[2]), and can be deployed independently of one another (e.g. convergent thickening, without extension, in *Xenopus* somites[2] and in zebrafish neurectoderm[4]; convergence alone in zebrafish lateral mesoderm[4]). A successful model of zebrafish epiboly will produce the observed tissue-level change and enable exploration of alternative cellular mechanisms. When a tissue changes shape, the cells within the tissue come to rest in new positions, but CE does not imply a particular cellular mechanism: it may involve cell rearrangement or cell shape change or a combination of both, and any cell movements or shape changes may be endogenously driven by the cells of the elongating tissue, or imposed on them by forces exerted on the tissue by neighboring tissues. In that context, when we speak of convergence of cells, we refer simply to their movement to arrive at the new position, not to mechanism. In any given model system, the cellular and molecular mechanism(s) of CE must be elucidated empirically[3,5], and can vary even among different regions of the same embryo, as in the zebrafish deep layer[4]; indeed, between different regions of the same cell[6–8].

Notably, zebrafish epiboly involves two distinct convergent extension processes, of two different types. Classically, convergent extension refers to the directional convergence of cells, moving laterally toward the midline of a tissue, while that tissue extends along its midline. Zebrafish gastrulation, which takes place between 50% and 100% epiboly (tailbud stage), involves the convergence of lateral cells toward the dorsal midline, and the narrowing and elongation of dorsal axial tissue – convergence and extension of the classical type[9] (Fig. 1b in main text). Inherently, this type of CE requires either a pair of free lateral edges that approach each other, such as in an explant[10], or differential thinning, where the ventral part of the tissue becomes depopulated in order to feed cells to the dorsal part ("dorsal convergence and ventral divergence" in *Xenopus*[2]; the "evacuation zone" in zebrafish[11,12]). Axial CE in zebrafish involves the deep cell layer and even the YSL; the EVL is the only part of the embryo that does not participate in it[11,13–15]. The shrinking of the leading edge of the vegetally extending blastoderm during epiboly represents a different variation on convergence and extension (and thinning[16]), which occurs when a circular or cylindrical tissue shrinks its circumference uniformly as the tissue elongates; then there is neither an identifiable midline, nor a lateral edge, and cells come together along the shrinking circumference, without lateral migration (Fig. 1c in main text). The cells may jostle around each other but net cell movement is in the direction of tissue elongation. In this mode of CE, the continuity of the deforming tissue around a central axis results in the narrowing of a cylinder, as in the secondary invagination and elongation of sea urchin archenteron[17,18]; the closing of a circular opening, as in vestibule closure during metamorphosis of the sea urchin *Heliocidaris erythrogramma*[19]; or the shrinking leading edge circumference of all participating tissues in zebrafish epiboly (including the EVL), after they pass the equator. Our model considers only epiboly and not axial development, and only the EVL, so our discussion of convergent extension refers exclusively to this second process.

1. Vogt, W. Gestaltungsanalyse am Amphibienkeim mit Örtlicher Vitalfärbung : II. Teil. Gastrulation und Mesodermbildung bei Urodelen und Anuren. *Wilhelm Roux Arch Entwickl Mech Org* **120**, 384–706 (1929).
2. Keller, R. E. Vital dye mapping of the gastrula and neurula of Xenopus laevis. II. Prospective areas and morphogenetic movements of the deep layer. *Dev Biol* **51**, 118–137 (1976).
3. Keller, R. E. The Cellular Basis of Gastrulation in Xenopus laevis: Active, Postinvolution Convergence and Extension by Mediolateral Interdigitation. *American Zoologist* **24**, 589–603 (1984).
4. Williams, M. L. K. & Solnica-Krezel, L. Cellular and molecular mechanisms of convergence & extension in zebrafish. *Curr Top Dev Biol* **136**, 377–407 (2020).
5. Keller, R. E. The cellular basis of epiboly: an SEM study of deep-cell rearrangement during gastrulation in Xenopus laevis. *J Embryol Exp Morphol* **60**, 201–234 (1980).
6. Williams, M., Yen, W., Lu, X. & Sutherland, A. Distinct Apical and Basolateral Mechanisms Drive Planar Cell Polarity-Dependent Convergent Extension of the Mouse Neural Plate. *Developmental Cell* **29**, 34–46 (2014).
7. Sun, Z. *et al.* Basolateral protrusion and apical contraction cooperatively drive Drosophila germ-band extension. *Nat Cell Biol* **19**, 375–383 (2017).
8. Huebner, R. J. & Wallingford, J. B. Coming to Consensus: A Unifying Model Emerges for Convergent Extension. *Developmental Cell* **46**, 389–396 (2018).
9. Warga, R. M. & Kimmel, C. B. Cell movements during epiboly and gastrulation in zebrafish. *Development* **108**, 569–580 (1990).
10. Keller, R. & Danilchik, M. Regional expression, pattern and timing of convergence and extension during gastrulation of Xenopus laevis. *Development* **103**, 193–209 (1988).

11. Kimmel, C. B., Warga, R. M. & Schilling, T. F. Origin and organization of the zebrafish fate map. *Development* **108**, 581–594 (1990).

12. Kimmel, C. B., Ballard, W. W., Kimmel, S. R., Ullmann, B. & Schilling, T. F. Stages of embryonic development of the zebrafish. *Dev Dyn* **203**, 253–310 (1995).

13. Kimmel, C. B. & Warga, R. M. Indeterminate cell lineage of the zebrafish embryo. *Dev Biol* **124**, 269–280 (1987).

14. Rohde, L. A. & Heisenberg, C.-P. Zebrafish gastrulation: cell movements, signals, and mechanisms. *Int Rev Cytol* **261**, 159–192 (2007).

15. D'Amico, L. A. & Cooper, M. S. Morphogenetic domains in the yolk syncytial layer of axiating zebrafish embryos. *Dev Dyn* **222**, 611–624 (2001).

16. Campinho, P. *et al.* Tension-oriented cell divisions limit anisotropic tissue tension in epithelial spreading during zebrafish epiboly. *Nat Cell Biol* **15**, 1405–14 (2013).

17. McClay, D. R., Warner, J., Martik, M., Miranda, E. & Slota, L. Gastrulation in the sea urchin. *Curr Top Dev Biol* **136**, 195–218 (2020).

18. Martik, M. L. & McClay, D. R. New insights from a high-resolution look at gastrulation in the sea urchin, Lytechinus variegatus. *Mech Dev* **148**, 3–10 (2017).

19. Minsuk, S. B. & Raff, R. A. Co-option of an oral-aboral patterning mechanism to control left-right differentiation: the direct-developing sea urchin Heliocidaris erythrogramma is sinistralized, not ventralized, by NiCl2. *Evol Dev* **7**, 289–300 (2005).

Legends to Supplementary Movies

Supplementary Movie 1. The model EVL, being stretched around the yolk from its initial configuration by an external force, without adding dynamic bond remodeling; mechanical coupling relationships between cells are fixed. Because individual bonds are elastic and neighbor relationships are fixed (precluding any possibility of cell rearrangement), the global behavior of the cell layer is likewise elastic. Note also the crumpling of the edge of the sheet (yellow particles) from its relatively straight path at the start of the simulation, to a highly folded structure at the end. After fully stretching the tissue around the yolk (at 0:08 in the video), we release the external forces, and the tissue deformation reverses, demonstrating the tissue elasticity. In contrast, a model of living tissue must be able to undergo permanent viscoelastic deformation, and cell rearrangement. (Tissue Forge has a programmable camera; in this and most subsequent movies, we have it automatically begin rotating to a vegetal position when any point on the EVL margin reaches a mean polar angle $\phi = 0.75\pi$.) (Corresponds to Fig. 3 in main text.)

Supplementary Movie 2. Model 1 with a permissive constraint ($\lambda = 1.0$), insufficient to maintain EVL integrity and prevent tearing of the tissue under tension.

Supplementary Movie 3. Model 1 with a stricter constraint ($\lambda = 3.75$, and here without cell division) generates epibolic movement resembling a living zebrafish embryo. However, epiboly progress becomes asynchronous at later stages, with the leading edge of the EVL shifting off-center relative to the vegetal pole, and finally ending in a protrusion toward the pole on one side. (Corresponds to Supplementary Figure 1.)

Supplementary Movie 4. Model 1 with cell division generates epibolic movement resembling a living zebrafish embryo. However, epiboly progress becomes asynchronous at later stages, with the leading edge of the EVL shifting off-center relative to the vegetal pole, and finally ending in a protrusion toward the pole on one side. (Corresponds to Fig. 5a in main text.)

Supplementary Movie 5. Model 1. Cells that have undergone division are labeled brown if located in the EVL margin, and light blue if located in the EVL interior. (Corresponds to Supplementary Figure 2.)

Supplementary Movie 6. Our enhanced, regulated model (Model 2), in which externally applied forces on the EVL leading edge are adjusted locally to ensure epiboly progresses synchronously around the whole embryo. (Corresponds to Fig. 5b in main text.)

Supplementary Movies 7–9. At higher λ values, EVL expansion and edge straightening slow down. In Supplementary Movie 7, $\lambda = 5$, epiboly completes, and the edge straightens, though more slowly than in Supplementary Movie 6 (where $\lambda = 3.75$). In Supplementary Movies 8 and 9, $\lambda = 6$ and $\lambda = 10$ respectively; EVL expansion slows to a halt and the leading edge never reaches the termination condition (99% epiboly), so these latter two were terminated manually. As λ is increased, the progressively lower frequency of bond-making and bond-breaking events throughout the EVL is visibly evident in the three movies, and is also reflected in the progressively larger margin cell populations (yellow particles) even at comparable stages of development. (The three movies correspond to Fig. 8g-i, respectively, in main text.)

Supplementary Movie 10. Lineage tracing. Model 2 with EVL margin cells labeled red at simulation initialization; all cells retain their color through the entire simulation. Cell division was disabled; therefore the observed widening of the labeled tier and the cell mixing are not due to cell division but only to cell rearrangement. (Corresponds to Fig. 10a in main text.)

Supplementary Movie 11. Lineage tracing, set up as in Supplementary Movie 10, but labeling tier 1 of internal EVL cells. (Corresponds to Fig. 10b in main text.)

Supplementary Movie 12. Lineage tracing, set up as in Supplementary Movie 10, but labeling tier 5 of internal EVL cells. (Corresponds to Supplementary Figure 4.)

Supplementary Movie 13. Lineage tracing, set up as in Supplementary Movie 10, but labeling a patch of EVL cells on one side of the embryo, adjacent to the leading edge. (Corresponds to Fig. 10c in main text.)

Supplementary Movie 14. Lineage tracing, set up as in Supplementary Movie 10, but labeling a patch of EVL cells on one side of the embryo, adjacent to the leading edge. In this instance, the patch separated completely from the leading edge. (Corresponds to Fig. 10d in main text.)

Supplementary Movie 15. Model 2, with a much stronger bond angle constraint along the margin (λ=10). Epiboly proceeds normally but the straightening of the EVL edge is delayed until later in epiboly, and the kinks along the edge appear rigid. Compare Supplementary Movie 6. (Corresponds to Fig. 11g in main text.)

Supplementary Movie 16. Laser-cut experiment (removal of external stretching force), Model 2, with a remodeling-disabled phase, during which the leading edge recoils a small distance and then stabilizes for about 5s of playback, followed by a remodeling-enabled phase, during which a much larger recoil occurs.

SUPPLEMENTARY FIGURES

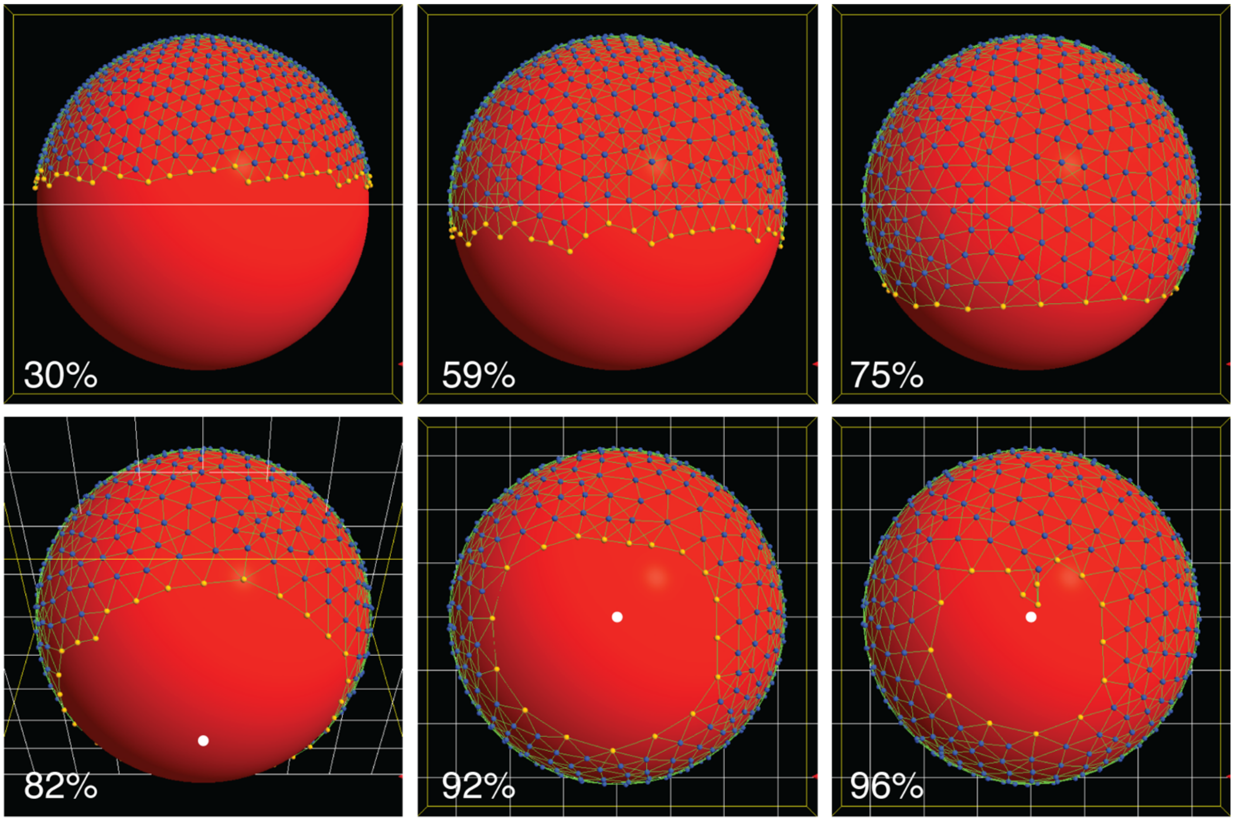

Supplementary Figure 1. Epiboly progression in Model 1 without cell division. See Supplementary Movie 3. (% epiboly stages scored and annotated, and vegetal pole marked, as in Fig. 5 in the main text.) As a consequence of stretching without cell division, individual cells acquire larger apical surface area as epiboly proceeds, as can be seen by comparing the cell packing density in the different panels.

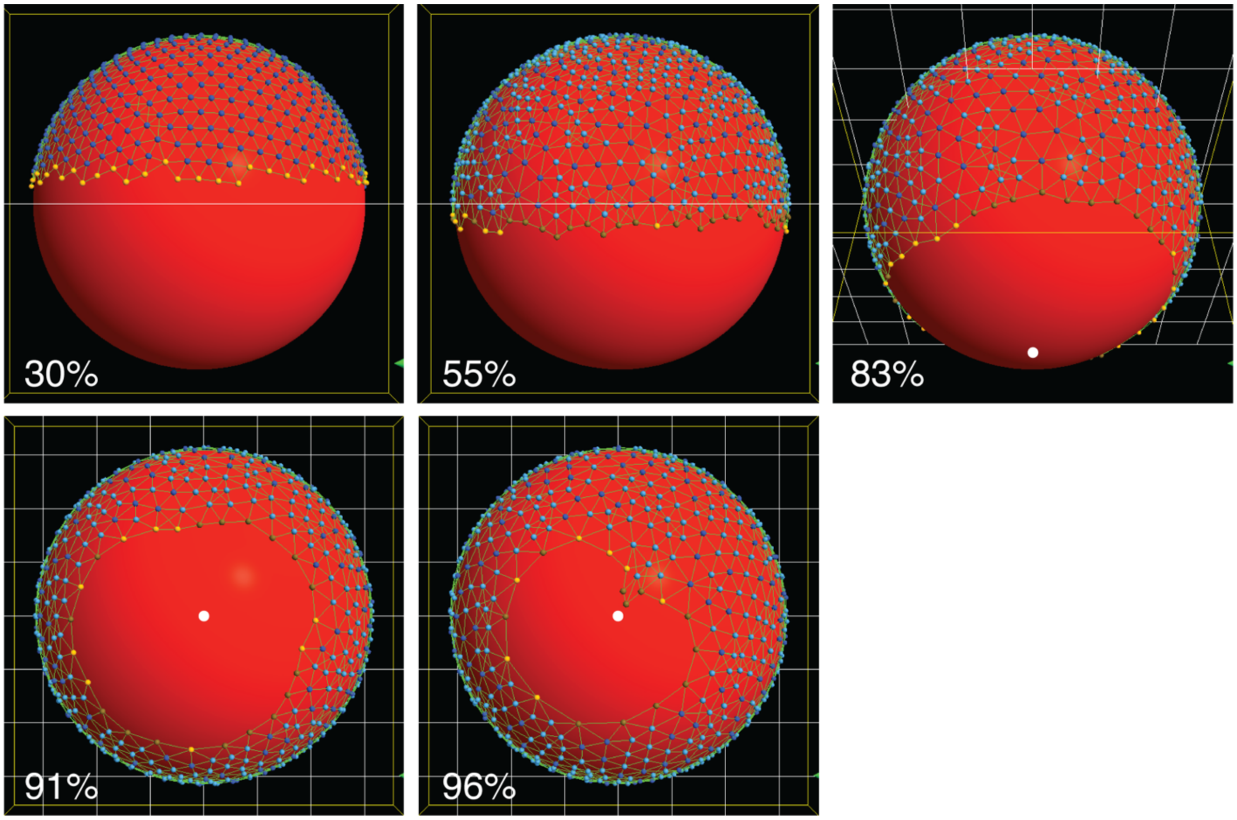

Supplementary Figure 2. Epiboly progression in Model 1 with cell divison, with color coding added to identify daughter cells. See Supplementary Movie 5. Cells that have divided are labeled brown if located in the EVL margin, and light blue if located in the EVL interior. The smaller size of the divided cells can be discerned, despite the lack of explicit cell boundaries, by their closer packing. All cell division takes place between 30% and 55% epiboly. (% epiboly stages scored and annotated, and vegetal pole marked, as in Fig. 5 in the main text.)

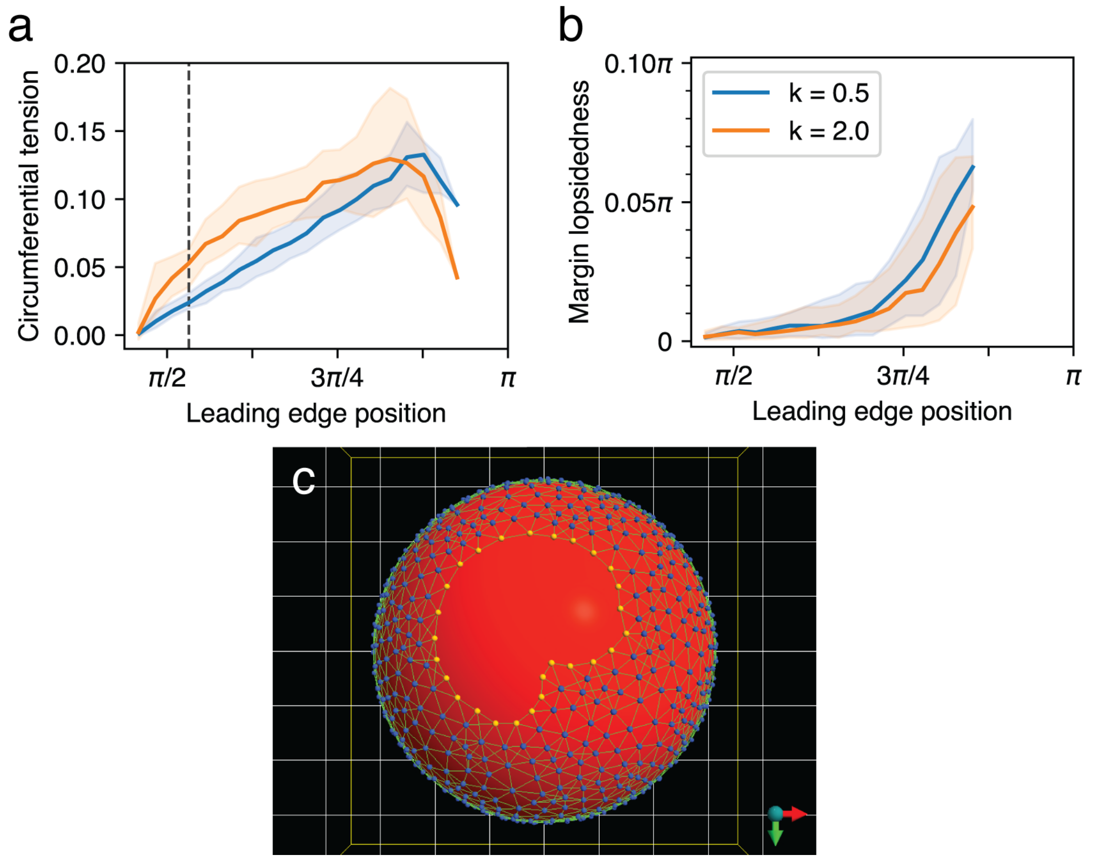

Supplementary Figure 3. In Model 1, increasing the spring constant, k, from 0.5 (as used throughout this paper except where noted) to 2.0 results in increased leading edge tension during most of epiboly but has only a small effect on lopsidedness and is not sufficient to ensure synchronous epiboly. Consensus plots of median values (N=32 replicates for each treatment) and 5–95 percentile ranges (shading). Legend in (b) applies to both plots.

a. Circumferential tension increases more quickly in the early part of epiboly when k = 2.0.

b. Median lopsidedness is slightly decreased when k = 2.0, but the ranges of variation are largely overlapping.

c. Final morphology of an example run with k = 2.0. Leading edge is lopsided and has the typical protrusion.

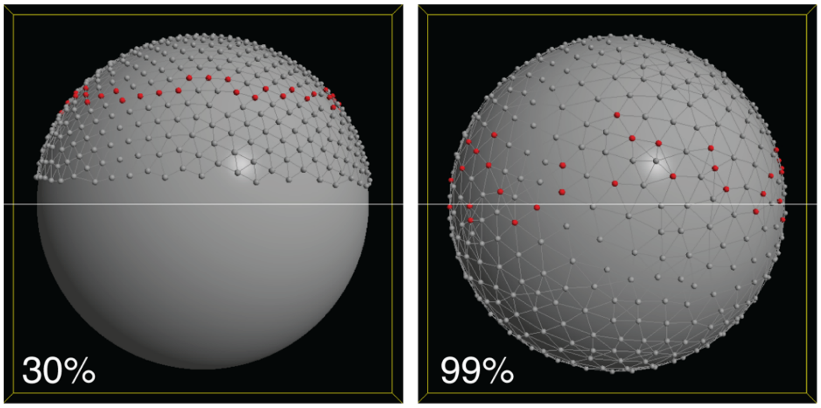

Supplementary Figure 4. Lineage tracing, set up as in Fig. 10 in the main text, but labeling tier 5 of internal EVL cells. 30% and 99% epiboly are shown. Camera rotation was disabled, to capture only the lateral view from beginning to end of the simulation, since the labeled cells never progress beyond the equatorial zone. See also Supplementary Movie 12.
